# Dimensional Structure of BERT-Estimated Emotion Representations in Adolescents With Autism Spectrum Disorder: An Exploratory Analysis of Clinical Narratives

**DOI:** 10.7759/cureus.106830

**Published:** 2026-04-11

**Authors:** Muneaki Kanno, Yuka Yoshida, Momoko Fujihashi, Nana Takahashi, Takuma Numazawa, Yunosuke Mizuno

**Affiliations:** 1 Department of Psychiatry, Yamagata University Faculty of Medicine, Yamagata, JPN

**Keywords:** autism specrum disorders, bert, emotion, natural language processing (nlp), principal component analysis (pca)

## Abstract

Background: Autism spectrum disorder (ASD) is characterized by impairments in emotion recognition and regulation. While natural language processing (NLP) offers a systematic approach to estimating emotions from clinical narratives, the latent dimensional structure of these estimated emotions in ASD remains insufficiently characterized using methodologically rigorous frameworks.

Objective: This exploratory study aimed to characterize the dimensional structure of model-estimated emotions in adolescents with ASD using a fine-tuned Japanese BERT model, while explicitly accounting for domain shift and compositional data constraints.

Methods: A Japanese BERT model (*tohoku-nlp/bert-large-japanese-v2*) was fine-tuned on the WRIME dataset (social media posts). The model was applied to 1,239 clinical sessions from 14 adolescents with ASD to generate eight-dimensional emotion probability vectors. To address the compositional nature of softmax outputs and the non-independence of repeated sessions, we performed a centered log-ratio (CLR) transformation followed by within-patient centering. Principal component analysis (PCA), with patient-level bootstrapping, was conducted to identify robust dimensions. In-domain validation was performed using 150 manually annotated clinical snippets.

Results: The model achieved 81.3% accuracy on the out-of-domain test set, though in-domain validation revealed performance variability across emotion categories (e.g., higher reliability for sadness than for trust). PCA identified two primary dimensions: PC1 (57.8% variance), characterized by a dominant sadness-driven internalizing axis, and PC2 (14.2% variance), representing a contrast between anticipation and aversive emotions (disgust/anger). These dimensions remained stable across bootstrap resampling.

Conclusions: This study demonstrates that transformer-based NLP, when combined with rigorous compositional data analysis, can elucidate latent emotional structures within clinical narratives. However, these patterns reflect a model-mediated representation space influenced by clinical documentation practices and domain-specific model characteristics. While providing a novel quantitative framework for psychiatric NLP, our findings emphasize the necessity of in-domain validation and cautious clinical interpretation.

## Introduction

Autism spectrum disorder (ASD) is a neurodevelopmental condition fundamentally characterized by persistent challenges in social communication and restricted, repetitive patterns of behavior. Beyond these core diagnostic features, impairments in emotion recognition and regulation are frequently reported and significantly impact social functioning and overall quality of life. Meta-analytic evidence suggests that individuals with ASD often exhibit reduced accuracy in recognizing basic emotions, such as happiness, sadness, fear, and disgust [1], while relying more heavily on maladaptive emotion regulation strategies [2]. Systematic characterization of emotional patterns in ASD is therefore essential to improve clinical understanding and support.

Theoretical frameworks, such as Plutchik’s Wheel of Emotions, provide a structured multidimensional perspective by organizing eight primary emotions into polar opposites with varying intensities [3]. This perspective is particularly relevant for examining the complex, mixed emotional states often encountered in psychiatric settings [4]. Recent advancements in transformer-based natural language processing (NLP) have enabled the operationalization of these theoretical models, allowing for the multidimensional estimation of emotion probabilities from clinical text. While prior research has demonstrated the utility of NLP in psychiatric contexts [5-8], many studies have focused on categorical classification or sentiment polarity, potentially overlooking the latent dimensional structure of emotional representations.

Furthermore, applying NLP models to clinical narratives presents significant methodological challenges, including the “domain shift” between social media training data and clinical documentation, as well as the statistical “closure problem” inherent in compositional softmax outputs. Addressing these factors is critical for ensuring the transparency and reliability of AI-based estimates in clinical research, as emphasized by the TRIPOD+AI reporting standards [9].

The primary objective of this exploratory study was to characterize the dimensional structure of model-estimated emotions in adolescents with ASD using clinical narratives extracted from electronic health records. To achieve this, we fine-tuned a Japanese BERT model to estimate multidimensional emotion vectors and applied a rigorous analytical pipeline, incorporating in-domain validation, centered log-ratio (CLR) transformation, and within-patient centering, to identify robust latent dimensions within the estimated emotion space. By distinguishing model-mediated representational structures from intrinsic clinical phenomena, this study seeks to provide a methodologically grounded foundation for the use of NLP in characterizing emotional dynamics in ASD.

This article was previously posted to the medRxiv preprint server on May 15, 2025 [10].

## Materials and methods

Study design and reporting standards

This exploratory study developed and applied a natural language processing (NLP) model to longitudinal electronic health records. The study reporting aligns with the TRIPOD+AI guidelines for AI-based prediction and estimation models to ensure transparency and reproducibility. A completed TRIPOD+AI checklist is provided in the Supplementary Material.

Datasets

This study used the WRIME dataset, a publicly available Japanese emotion corpus comprising 43,200 short social media posts. Each post was annotated according to Plutchik’s eight basic emotions (joy, sadness, anticipation, surprise, anger, fear, disgust, and trust) [11]. Emotion intensity was rated on a four-point scale (none, weak, medium, strong) by three independent readers. Averaged reader-based annotations were used as target labels to capture continuous multidimensional emotion intensity. The dataset was divided into 40,000 training posts, 1,200 validation posts, and 2,000 test posts.

Model training and evaluation framework

The Japanese transformer model tohoku-nlp/bert-large-japanese-v2 (Hugging Face) was fine-tuned to output eight-dimensional continuous emotion probability vectors. The model was optimized using a cross-entropy loss function over normalized emotion distributions, with target representations derived from the aggregated reader-annotated intensity vectors. Fine-tuning was conducted with a batch size of 32, a learning rate of 2 × 10^-5^ using a linear scheduler with a 10% warmup period, and four training epochs under mixed-precision (FP16) settings. Model performance was evaluated at the end of each epoch, and the checkpoint from the second epoch was selected as the final model because it achieved the minimum MAE on the validation set. Performance on the out-of-domain (WRIME) test set was evaluated using two distinct frameworks: single-label classification accuracy (comparing the model’s argmax emotion with the annotators' predominant label) and vector-level agreement. The latter was measured by calculating the cosine similarity between the model-generated probability vector and the averaged reader-annotated intensity vector for each of the 2,000 test posts, and subsequently computing the overall mean.

Participants and clinical text processing

Fourteen adolescents (10 male, 4 female) diagnosed with ASD according to DSM-5 criteria were retrospectively identified. Electronic health records from their initial visits between 2011 and 2017 through December 2024 were exported. The sample size was determined by the available clinical cohort for this exploratory analysis, and no a priori sample size calculation was performed.

Patient speech was extracted from routine outpatient free-text documentation using rule-based string processing based on speaker tags. To ensure data quality, the extracted text was manually verified by the authors to remove misattributed segments, such as speech from family members or clinicians. The final dataset consisted of complete session-level text entries; therefore, no missing data imputation procedures were required.

In-domain validation

Because the model was trained on social media text, an in-domain validation study was conducted to address potential domain shift in clinical narratives. A stratified subset of 150 clinical text snippets was randomly sampled across different patients and time points. Two independent annotators assigned intensity scores (0-3) for the eight Plutchik emotions. We evaluated both inter-rater reliability and model-annotator agreement using Spearman’s rank correlation coefficient (ρ), quadratic weighted kappa (QWK), and mean absolute error (MAE).

Feature extraction and statistical analyses

For each clinical session, the fine-tuned BERT model generated an eight-dimensional softmax vector representing emotion probabilities. Because softmax outputs constitute closed compositional data (summing to one), analyzing raw proportions via principal component analysis (PCA) can induce spurious correlations due to the closure constraint. To address this, we applied a centered log-ratio (CLR) transformation to map the compositional data into an unconstrained Euclidean space.

Furthermore, to account for the non-independence of repeated sessions nested within individual patients, we applied within-patient centering by subtracting each patient’s mean CLR-transformed emotion vector from their session-level vectors. PCA was then performed on these CLR-transformed, within-patient-centered data. To ensure robustness and account for clustering at the participant level, patient-level bootstrap resampling was conducted to estimate 95% confidence intervals (CIs) for both PCA loadings and explained variance. All analyses were conducted in Python.

Ethical considerations

This retrospective study was conducted at a single medical institution and approved by the Ethical Review Committee of the Yamagata University Faculty of Medicine (Approval No. 2023-159). In accordance with institutional and national ethical guidelines, the requirement for written informed consent was waived, and an opt-out consent procedure was implemented.

All data were analyzed in a secure offline environment on an access-restricted local device managed by the first author. No identifiable information was transferred outside the institution or uploaded to external cloud-based services.

## Results

Out-of-domain and in-domain model performance

**Figure 1:**
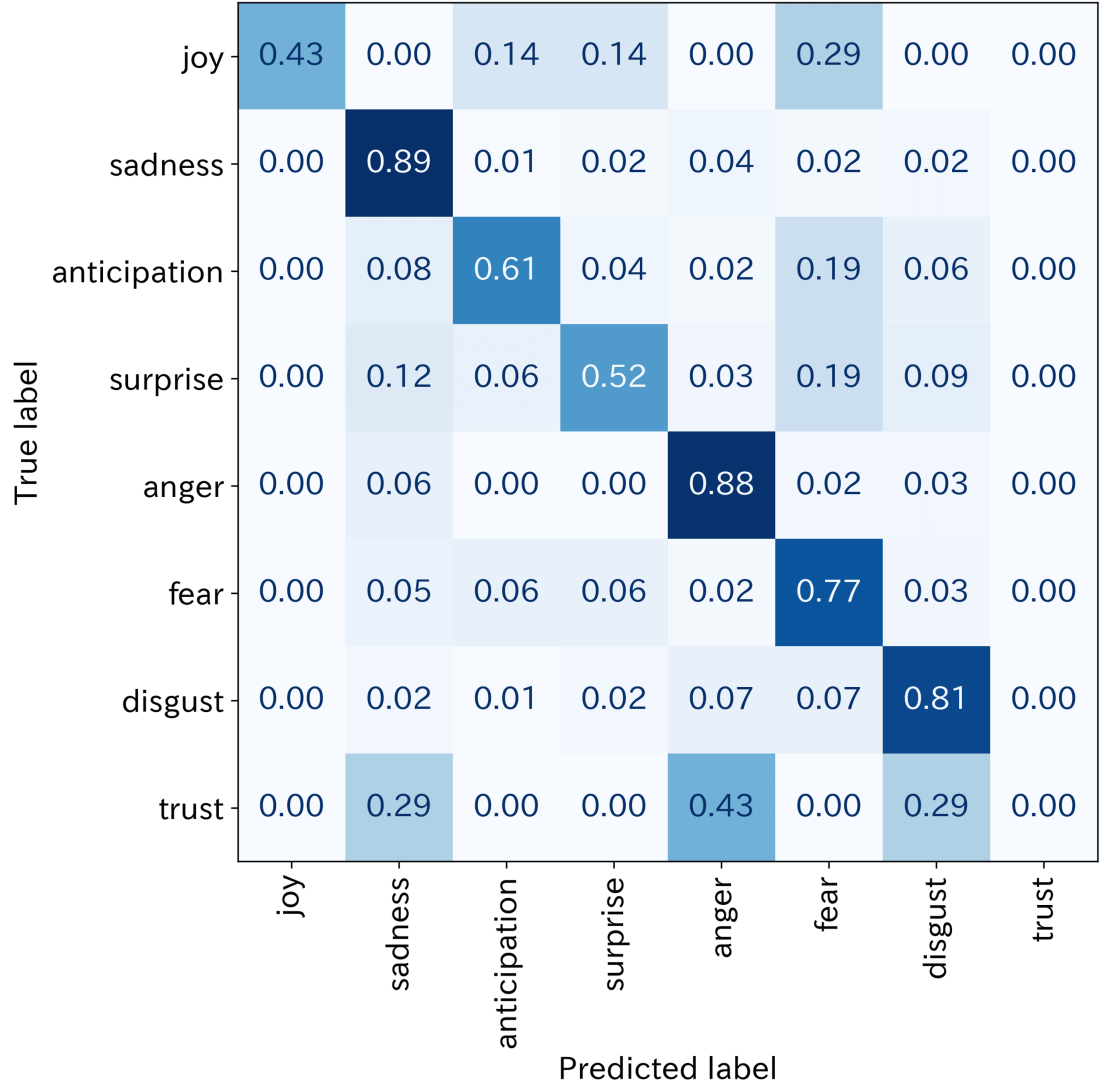
Confusion matrix of the fine-tuned model based on tohoku-nlp/bert-large-japanese-v2 The confusion matrix shows classification results for the eight basic emotions in the WRIME test dataset (n = 2,000). Diagonal elements represent correct classifications, whereas off-diagonal elements indicate misclassifications between emotion categories.

On the held-out WRIME test dataset, the model achieved a single-label accuracy of 81.3% and a mean vector-level cosine similarity of 92.2%, indicating strong out-of-domain performance. The confusion matrix shows classification results for the eight basic emotions in the WRIME test dataset (n = 2,000) (Figure 1).

However, the in-domain validation on the 150-snippet clinical subset revealed domain-specific variability. Inter-rater agreement for the manual annotations ranged from fair to substantial; agreement was strongest for anger (QWK = 0.684, ρ = 0.684) and disgust (QWK = 0.645, ρ = 0.688), moderate for sadness (QWK = 0.571, ρ = 0.578), and relatively lower for joy (QWK = 0.373, ρ = 0.253) and trust (QWK = 0.359, ρ = 0.399) (Table 1). This confirms the feasibility of the annotation framework, particularly for identifying prominent aversive and internalizing emotions in clinical text.

**Table 1:** Inter-rater agreement for manual emotion annotation (n = 150) Values represent the agreement between two independent human annotators who evaluated the intensity of Plutchik’s eight basic emotions across 150 clinical text snippets. Annotators assigned intensity scores on a 4-point scale ranging from 0 to 3. The reported p-values indicate the statistical significance of Spearman’s rank correlation coefficient (ρ). Mean Absolute Error (MAE) was calculated using intensity scores scaled to a 0 to 1 range. Emotions are listed in descending order of QWK.

Emotion	Quadratic Weighted Kappa (QWK)	Spearman’s ρ	p-value	Mean Absolute Error (MAE)
Anger	0.684	0.684	<0.001	0.138
Disgust	0.645	0.688	<0.001	0.189
Sadness	0.571	0.578	<0.001	0.18
Surprise	0.491	0.35	<0.001	0.062
Anticipation	0.452	0.419	<0.001	0.113
Fear	0.426	0.559	<0.001	0.262
Joy	0.373	0.253	0.002	0.144
Trust	0.359	0.399	<0.001	0.127

In parallel, the agreement between the model’s estimated emotion probabilities and the manual annotations varied substantially across emotion categories. The highest mean model-annotator agreement was observed for sadness (mean QWK = 0.492, mean ρ = 0.431), followed by moderate agreement for joy (mean QWK = 0.389), anticipation (mean QWK = 0.380), and disgust (mean QWK = 0.270). Conversely, agreement was near zero for trust (mean QWK = 0.000, mean ρ = -0.049) and exhibited weak or negative correlations for anger (mean QWK = 0.059, mean ρ = -0.137) (Table 2). The overall mean agreement across all emotions was modest (Annotator 1: mean QWK = 0.266; Annotator 2: mean QWK = 0.241) (Table 3). These findings indicate that while the model captures internalizing dimensions such as sadness with reasonable fidelity, its performance does not transfer uniformly across all emotion categories in the clinical domain, highlighting specific degradation in dimensions like trust and anger due to domain shift.

**Table 2:** Agreement between model-estimated emotion probabilities and manual annotations (n =150) Values represent the mean agreement metrics between the model-estimated emotion probabilities and the manual annotations across the 150 clinical text snippets. The agreement metrics were calculated separately for each of the two independent human annotators and subsequently averaged. Mean Absolute Error (MAE) was calculated by comparing the model's output probabilities with the human intensity scores, which were scaled to a 0 to 1 range for direct comparison. Emotions are listed in descending order of Mean QWK.

Emotion	Mean Quadratic Weighted Kappa (QWK)	Mean Spearman’s ρ	Mean Absolute Error (MAE)
Sadness	0.492	0.431	0.225
Joy	0.389	0.246	0.141
Anticipation	0.38	0.248	0.156
Disgust	0.27	0.3	0.265
Surprise	0.223	0.062	0.125
Fear	0.216	0.121	0.284
Anger	0.059	-0.137	0.228
Trust	0	-0.049	0.134

**Table 3:** Overall agreement metrics between the emotion estimation model and individual annotators Values represent the mean agreement metrics averaged across all eight Plutchik emotion categories for each independent annotator based on the 150 clinical text snippets. Mean Absolute Error (MAE) was calculated using scaled intensity scores from 0 to 1.

Annotator	Mean Quadratic Weighted Kappa (QWK)	Mean Spearman’s ρ	Mean Absolute Error (MAE)
Annotator 1	0.266	0.181	0.17
Annotator 2	0.241	0.125	0.219

Participant characteristics and emotion profile estimation

The clinical sample consisted of 14 adolescents with ASD, followed for a median duration of 11.5 years with a median of 86.5 visits per participant (Table 4). The model generated 1,239 session-level emotion probability vectors from the clinician-documented patient speech. Across all sessions, sadness was the most frequently predominant estimated emotion, while trust was the least frequent (Table 5).

**Table 4:** Participant demographic and clinical characteristics (n = 14) Values are presented as mean ± standard deviation (median, interquartile range). ADHD: attention-deficit/hyperactivity disorder.

Characteristics	n = 14
Sex, n (%)	Male 10 (71%); Female 4 (29%)
Age at first visit, years	12.7 ± 4.1 (12.5, 11.0–15.3)
Age at last visit, years	24.9 ± 3.2 (24.0, 22.3–27.8)
Duration of outpatient follow-up, years	10.4 ± 2.4 (11.5, 8.3–12.0)
Number of outpatient visits	88.5 ± 25.0 (86.5, 72.5–104.8)
Full-scale IQ	91.2 ± 17.9 (90.5, 77.0–101.8)
ADHD, n (%)	7 (50%)
Major depressive disorder, n (%)	2 (14%)
Obsessive–compulsive disorder, n (%)	1 (7%)

**Table 5:** Mean emotion probabilities and number of sessions in which each emotion was predominant (n = 1,239) Values represent softmax-derived emotion probabilities estimated from clinician-documented patient speech. Predominant emotion was defined as the emotion with the highest probability within each of the 1,239 outpatient sessions.

Emotion	Mean Probability (±SD)	Number of Sessions Where Emotion Was Predominant
Joy	0.103 ± 0.116	154
Sadness	0.266 ± 0.240	482
Anticipation	0.151 ± 0.164	242
Surprise	0.096 ± 0.096	77
Anger	0.054 ± 0.047	3
Fear	0.143 ± 0.117	146
Disgust	0.136 ± 0.146	135
Trust	0.051 ± 0.040	0

Principal component analysis of latent emotion structure

PCA conducted on the CLR-transformed and within-patient centered data revealed a robust low-dimensional structure, with the first four principal components collectively accounting for approximately 90.1% of the total variance (Table 6). 

**Table 6:** Explained variance of principal components with bootstrap confidence intervals (centered log-ratio-transformed, within-patient centered) Explained variance of principal components derived from CLR-transformed emotion compositions after within-patient centering, with 95% confidence intervals estimated by patient-level bootstrap resampling. The first two components consistently accounted for the majority of the variance (mean 74.5%), with narrow confidence intervals indicating stable low-dimensional structure. These findings support the robustness of the principal component solution and suggest that the observed emotional organization is not solely driven by sampling variability or clustering effects.

Principal Component	Explained Variance (%)	95% CI
PC1	57.8	52.3–62.6
PC2	16.7	14.3–20.2
PC3	9.1	7.3–11.1
PC4	6.5	5.5–8.0

The first principal component (PC1) explained the majority of the variance at 57.8% (95% CI, 52.3-62.6) and was primarily defined by a strong positive loading for sadness (0.676; 95% CI, 0.599-0.733), functioning as a dominant internalizing dimension. The second principal component (PC2) explained 16.7% of the variance (95% CI, 14.3-20.2) and was characterized by a contrast involving anticipation (0.582; 95% CI, 0.392-0.692) in opposition to aversive emotions, specifically anger (-0.520; 95% CI, -0.560 to -0.460) and disgust (-0.499; 95% CI, -0.656 to -0.346) (Table 7). The loading structure is visualized in Figure 2.

**Table 7:** Bootstrap confidence intervals for principal component analysis loadings (centered log-ratio-transformed, within-patient centered) Principal component loadings for CLR-transformed emotion compositions after within-patient centering, with 95% confidence intervals estimated by patient-level bootstrap resampling. The first principal component (PC1) represents an aversive-internalizing emotional configuration characterized by sadness, fear, and disgust versus joy, trust, anticipation, and anger. The second principal component (PC2) reflects a contrast between anticipation and disgust/anger. Loadings whose confidence intervals do not cross zero indicate stable contributions across bootstrap samples.

Emotion	PC1 Loading (95% CI)	PC2 Loading (95% CI)
Sadness	0.676 (0.599 to 0.733)	0.234 (0.108 to 0.324)
Disgust	0.309 (0.221 to 0.407)	-0.499 (-0.656 to -0.346)
Fear	0.266 (0.220 to 0.303)	0.145 (0.054 to 0.245)
Joy	-0.361 (-0.396 to -0.320)	0.208 (0.143 to 0.275)
Trust	-0.345 (-0.382 to -0.305)	-0.152 (-0.261 to -0.039)
Anticipation	-0.254 (-0.290 to -0.218)	0.582 (0.392 to 0.692)
Anger	-0.247 (-0.297 to -0.192)	-0.520 (-0.560 to -0.460)
Surprise	-0.044 (-0.100 to 0.022)	0.003 (-0.094 to 0.159)

Subsequent components contributed progressively less to the overall structural variance, with the third (PC3) and fourth (PC4) principal components explaining 9.1% (95% CI, 7.3-11.1) and 6.5% (95% CI, 5.5-8.0), respectively. The robust confidence intervals and the stability of these components, particularly PC1 and PC2, across patient-level bootstrap resamples indicate that the observed emotional structure was not solely driven by sampling variability, compositional artifacts, or between-patient differences. In contrast, the factor loadings for PC3 and PC4 were not statistically robust, as their 95% confidence intervals derived from patient-level bootstrapping encompassed zero for all emotion categories.

## Discussion

This exploratory study investigated the dimensional structure of estimated emotions in adolescents with ASD by applying a fine-tuned Japanese BERT model to longitudinal clinical narratives. By utilizing CLR transformation to address compositional data constraints and within-patient centering to account for repeated measures, we identified a robust low-dimensional emotional architecture. This structure was primarily characterized by a dominant sadness-related dimension (PC1) and a secondary contrast between anticipation and aversive emotions, specifically anger and disgust (PC2).

Model performance and domain shift

While the NLP model demonstrated robust performance on the out-of-domain social media dataset, our in-domain validation revealed critical insights into domain shift and task complexity. Notably, human annotators achieved substantial agreement in identifying prominent aversive emotions such as anger and disgust, and moderate agreement for sadness, whereas agreement was notably weaker for positive or subtle emotions like joy and trust. This suggests that certain emotions are inherently more ambiguous or less explicitly verbalized in clinical documentation.

Crucially, the agreement between the model and human annotators did not transfer uniformly across emotion categories. The model achieved its highest in-domain reliability for sadness, which reinforces the validity of our primary PCA finding (PC1). Conversely, the model exhibited near-zero agreement for trust and weak-to-negative correlations for anger. This discrepancy, where humans can reliably detect anger but the model cannot, strongly underscores the presence of domain shift. The linguistic expressions of anger and trust in psychiatric clinical notes likely differ fundamentally from those in the WRIME social media corpus. Consequently, the outputs of this model must be interpreted as in-domain-validated but imperfect, model-mediated emotion estimates rather than absolute emotional truths.

Latent structure of emotion space

The PCA revealed that PC1, accounting for over half of the structural variance, was dominated by sadness. This is conceptually consistent with prior literature reporting elevated internalizing symptoms and a high risk of depressive features in autistic adolescents [12-17]. However, because our findings reflect the statistical structure of model-estimated representations rather than validated clinical measures of mood or formal diagnoses, the association between PC1 and depressive psychopathology should be regarded as strictly hypothesis-generating.

Furthermore, PC2 represented a robust contrast between anticipation and aversive emotions, prominently featuring anger and disgust. This configuration diverges from the polar-opposite relationships proposed in Plutchik’s circumplex model, wherein disgust typically opposes trust, and anticipation opposes surprise [18]. Although this deviation is conceptually consistent with the known intolerance of uncertainty and preference for sameness in individuals with ASD [19-22], we posit that it reflects a combination of methodological and domain-specific factors. Specifically, the near absence and poor model estimation of "trust" in our clinical sample likely forced a reconfiguration of the emotional space. When transformed via CLR to resolve compositional closure, anticipation emerged in opposition to a cluster of negative, aversive emotions (anger and disgust). Therefore, the identified structure represents a context-dependent, model-mediated representational geometry rather than an intrinsic remapping of human emotion.

Limitations and future directions

Several methodological limitations must be transparently acknowledged. First, emotional signals were inferred from clinician-documented patient speech. Despite rigorous manual verification to exclude misattributions, these narratives inherently contain clinician paraphrasing, summarization, and stylistic influences, introducing documentation bias.

Second, the softmax-derived emotion vectors were treated as relative intensity estimates within a compositional framework. While we used CLR transformation to mitigate the closure problem, we did not apply formal probability calibration techniques (e.g., temperature scaling or expected calibration error). Miscalibration in specific emotion dimensions could still subtly influence the observed covariance structure.

Third, we did not perform convergent validity testing to link the PCA-derived dimensions (e.g., PC1) to independent clinical variables, such as confirmed depressive episodes, treatment modifications, or standardized rating scales. The clinical significance of these dimensions remains provisional.

Finally, the sample size was relatively small and derived from a single institution, limiting generalizability. The sample size was determined by available data and is insufficient for stable population-level inference.

Future studies should aim to replicate these findings in larger, multicenter cohorts utilizing mixed-effects frameworks, incorporating formal model calibration, and validating NLP-derived emotional dimensions against direct, standardized clinical assessments.

## Conclusions

This study demonstrates that transformer-based NLP, when combined with rigorous compositional data analysis and an awareness of domain shift, can elucidate latent emotional structures within the clinical narratives of adolescents with ASD. We identified a prominent sadness-driven internalizing dimension and an anticipation-aversive contrast. However, these patterns reflect a model-mediated space heavily influenced by clinical documentation practices and domain-specific model degradation. While offering a novel quantitative framework for psychiatric NLP research, our findings emphasize the necessity of in-domain validation, methodological transparency, and cautious clinical interpretation.
